# Huntington's Disease: A Case Report of a Patient With a Positive Family History

**DOI:** 10.7759/cureus.92119

**Published:** 2025-09-12

**Authors:** Piotr Nowakowski, Grazyna Waska, Elwira Misztela-Lisiecka, Magdalena Miernik-Skrzypczak, Anna Strozak

**Affiliations:** 1 Internal Medicine, Municipal Hospital in Gliwice, Gliwice, POL; 2 Internal Medicine, Specialist Hospital No. 1 in Bytom, Bytom, POL; 3 Internal Medicine, Pabianice Medical Center, Pabianice, POL; 4 Pulmonology and Hematology, Lower Silesian Oncology Center, Wrocław, POL; 5 Internal Medicine, Dr. Anna Gostynska Wolski Hospital, Warsaw, POL

**Keywords:** autosomal dominant disorder, cag triplet repeat, huntingtin (htt), huntington's disease (hd), neurodegenerative disease, positive family history

## Abstract

Huntington's disease (HD) is an autosomal dominant neurodegenerative disorder caused by an expanded cytosine-adenine-guanine (CAG) trinucleotide repeat in the huntingtin (HTT) gene, typically presenting with progressive motor, cognitive, and psychiatric symptoms between the ages of 30 and 50 years. This study presents the clinical features of a 48-year-old woman with a three-year history of progressive memory impairment, concentration difficulties, and weakness. Neurological examination revealed psychomotor slowing, mild cognitive impairment, right-sided apparent weakness due to impaired motor coordination, right-sided hypoesthesia, hyperreflexia, mild dysarthria, extrapyramidal gait disturbances, and occasional choreiform movements. Laboratory tests excluded infectious and metabolic causes, and brain magnetic resonance imaging (MRI) showed no acute lesions. Electroencephalography (EEG) revealed focal slow waves in the left posterior region without epileptiform discharges, and neuropsychological assessment indicated borderline dementia with depressive-anxiety symptoms. Genetic testing confirmed a pathogenic CAG repeat expansion in the HTT gene (47 repeats in the affected allele), establishing the diagnosis of HD according to European Molecular Quality Network (EMQN) guidelines. This case emphasizes the importance of integrating clinical evaluation, neuropsychological assessment, and genetic testing in patients with progressive cognitive and motor symptoms, particularly when there is a positive family history.

## Introduction

Huntington's disease (HD) is a progressive, autosomal dominant neurodegenerative disorder caused by an expanded cytosine-adenine-guanine (CAG) trinucleotide repeat in the huntingtin (HTT) gene, resulting in production of mutant huntingtin (mHTT) protein and selective neuronal vulnerability most pronounced in the striatum and cerebral cortex [1,2]. First clinically characterized in the 19th century, HD manifests with a triad of motor, cognitive, and psychiatric disturbances; motor features classically include chorea and dystonia, whereas cognitive decline-often affecting executive functions and verbal learning-and a broad spectrum of psychiatric symptoms frequently precede or accompany motor onset [2-4]. Age at onset is inversely correlated with CAG repeat length, with full penetrance typically seen at greater than or equal to 40 repeats and reduced penetrance between 36 and 39 repeats; anticipation (especially with paternal transmission) is an established phenomenon driven by intergenerational expansion of repeats [2,3].

Epidemiological studies show notable geographic and ethnic variation in prevalence, with higher rates reported in populations of European ancestry compared with many Asian and African populations [1,2]. Advances in molecular genetics since the early 1990s have made genetic testing the diagnostic gold standard and have enabled accurate confirmation of HD in symptomatic individuals as well as predictive testing in at-risk relatives [1,3].

Despite a well-defined genetic cause, the precise molecular mechanisms linking mHTT to selective neuronal dysfunction remain incompletely understood, and current licensed therapies are symptomatic rather than disease-modifying. However, recent translational progress, including strategies to lower mHTT expression and other targeted approaches, has reinvigorated therapeutic research and clinical trials in HD [1,3,4].

The purpose of this report is to present a rare early-stage HD case in which cognitive symptoms dominated and motor findings were mild and asymmetric, underscoring the role of neuropsychological evaluation in diagnosis and the value of integrating family history into early clinical suspicion. Highlighting such symptoms may assist not only neurologists but also psychiatrists, general practitioners, and other clinicians in recognizing HD at an earlier stage.

## Case presentation

A 48-year-old woman was admitted to the Neurology Department for diagnostic evaluation due to a three-year history of progressive memory impairment, concentration difficulties, and generalized weakness. Her medical history included right shoulder joint injury under orthopedic care and urinary incontinence requiring pads. Family history was significant for HD in her mother and brother.

On neurological examination, the patient was alert but exhibited psychomotor slowing, mild cognitive impairment, and decreased responsiveness. There was hypoesthesia and mild weakness of the right upper and lower limbs (grade IV on Lovett scale [5]), hyperreflexia on the right side, and mild dysarthria. No Babinski sign was observed. Gait assessment revealed extrapyramidal features, and occasional mild choreiform movements of the trunk and limbs were noted.

**Figure 1:**
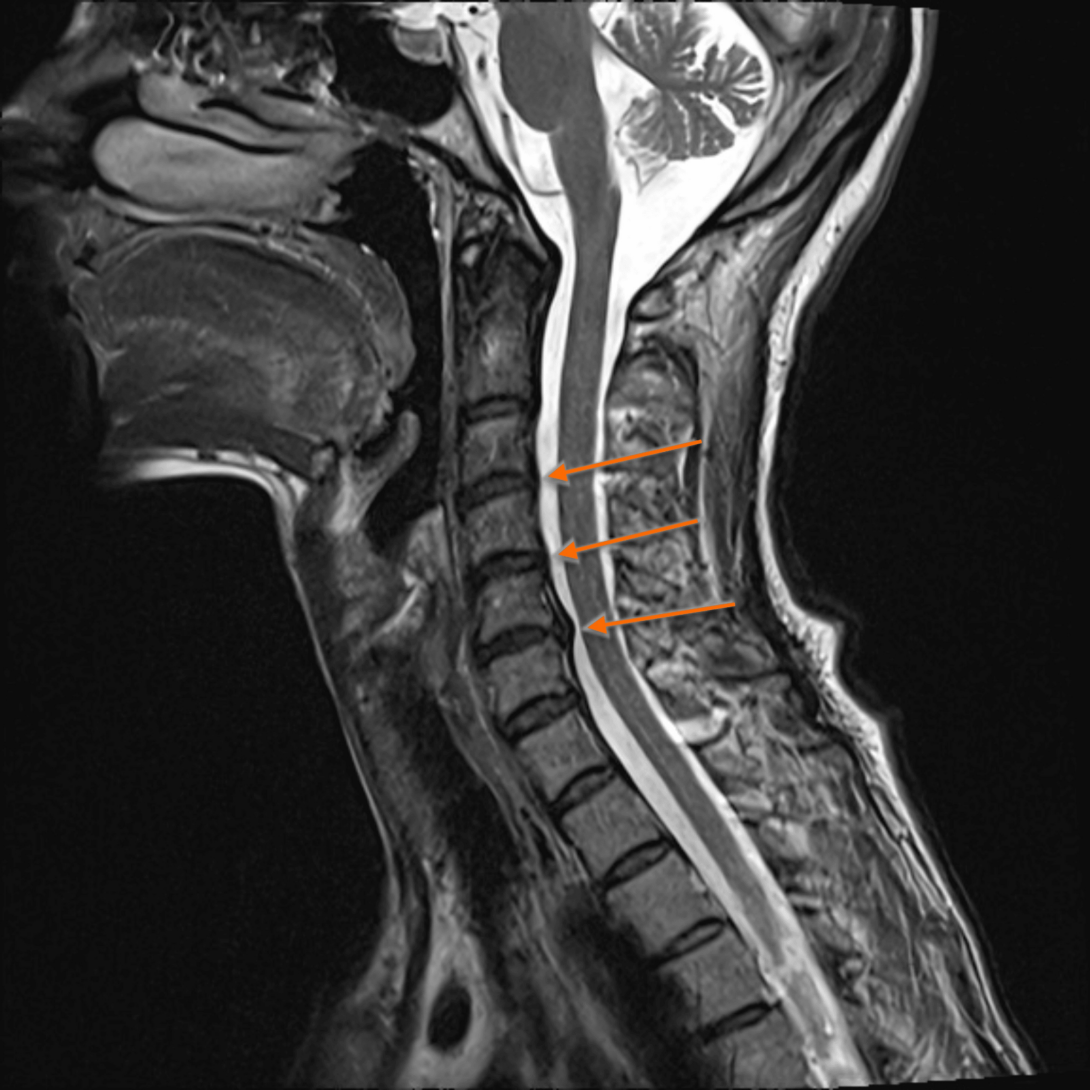
T2-weighted sagittal MRI of the cervical spine demonstrating moderate degenerative disc changes at C3-C4 and C4-C5, and disc bulge at C5-C6 (orange arrows). MRI: magnetic resonance imaging.

**Figure 2:**
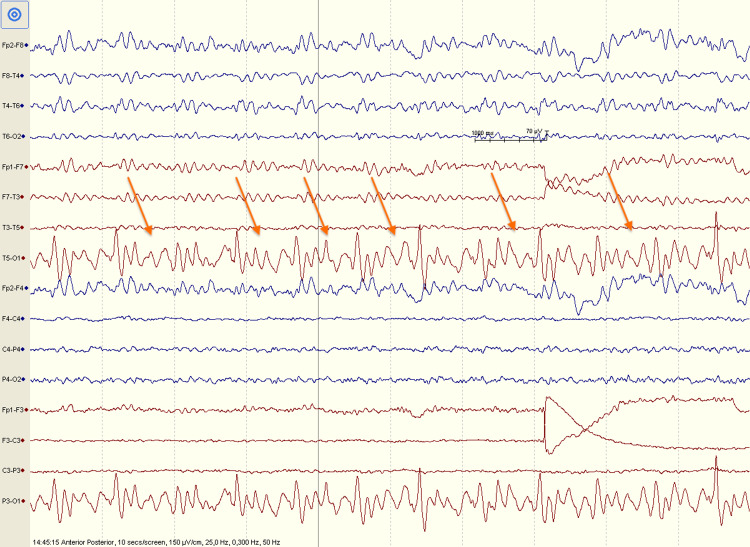
EEG recordings showing frequent slow theta waves in the left occipital region (orange arrows). EEG: electroencephalogram.

Magnetic resonance imaging (MRI) of the brain revealed no acute abnormalities. Cervical spine MRI showed degenerative disc disease without spinal cord compression (Figure 1). Laboratory tests were negative for human immunodeficiency virus (HIV), syphilis, and Borrelia infections. Vitamin B12 levels were at the lower limit of normal. Abdominal ultrasound was unremarkable. Electroencephalography (EEG) showed frequent slow waves in the left posterior regions without epileptiform discharges (Figure 2). Neuropsychological assessment indicated borderline dementia with depressive-anxiety symptoms, confirmed by psychological testing. The test results are presented in Table 1.

**Table 1:** Summary of test results. HD: Huntington's disease, HTT: huntingtin, CAG: cytosine-adenine-guanine, MRI: magnetic resonance imaging, EEG: electroencephalography, ±: plus or minus.

Test/investigation	Result	Interpretation
Brain MRI	No acute lesions; possible basal ganglia atrophy	Consistent with HD
Cervical spine MRI	Degenerative disc disease; no spinal cord compression	Not related to HD
EEG	Frequent slow waves in left posterior regions; no epileptiform discharges	Nonspecific finding
Neuropsychological assessment	Borderline dementia; attention and executive dysfunction	Consistent with HD
Genetic testing (HTT gene)	Pathogenic expansion: 47 ± 3 CAG repeats in affected allele	Diagnostic for HD

**Figure 3:**
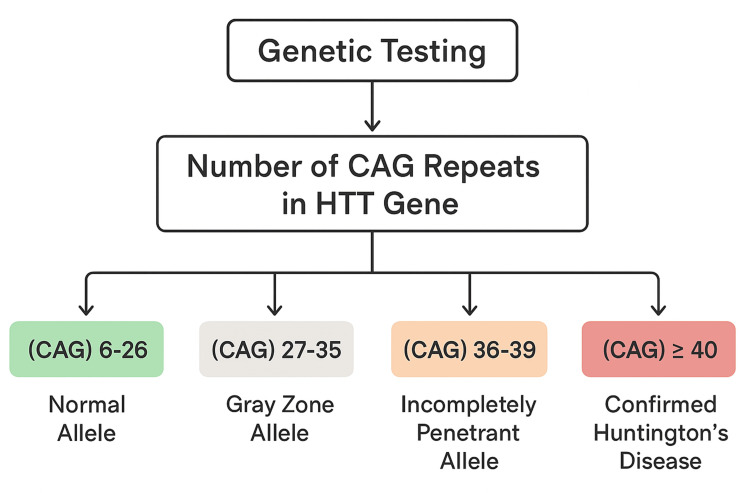
Interpretation of CAG repeat testing in the HTT gene (according to EMQN 2012 recommendations). HTT: huntingtin, CAG: cytosine-adenine-guanine, ≥: greater than or equal to, EMQN: European Molecular Quality Network.

Given the positive family history, genetic testing for the HTT gene was performed (Figure 3). Results revealed a pathogenic expansion of CAG trinucleotide repeats: 47 ± 3 in the affected allele and 17 ± 1 in the normal allele, confirming the diagnosis of HD according to European Molecular Quality Network (EMQN) 2012 recommendations.

## Discussion

HD is a progressive, autosomal dominant neurodegenerative disorder marked by cognitive decline, psychiatric disturbances, and motor dysfunction. Increasing evidence shows that cognitive and motivational deficits often emerge well before overt motor symptoms, highlighting the significance of early neuropsychological evaluation in HD gene carriers [6].

In this case, the patient's progressive memory impairment, psychomotor slowing, urinary incontinence, subtle choreiform movements, and mild right-sided weakness, with a positive family history, suggested early-stage HD. The genetic finding of 47 CAG repeats confirmed the diagnosis in line with established guidelines. Importantly, these cognitive symptoms predated the more recognizable motor signs, which had only mild asymmetry-a feature increasingly reported in HD's early stages [7,8].

Neuroimaging and neuropathological studies have revealed that asymmetric striatal and cortical degeneration can occur in HD, potentially accounting for unilateral motor manifestations [8]. Such findings challenge the assumption that motor features in HD are always symmetric from onset. Additionally, preclinical models such as the zQ175 mouse demonstrate that complex cognitive and motivational impairments emerge before motor dysfunction, aligning with the human prodromal phase observed in this patient [9].

The initial differential diagnosis effectively ruled out treatable causes, such as vitamin B12 deficiency, structural brain abnormalities, and infections. This comprehensive approach is critical, as non-motor symptoms may dominate early presentations and mimic other neurological or psychiatric disorders.

From a clinical perspective, this case reinforces the importance of integrating family history into early diagnostic consideration, performing targeted genetic testing, and initiating multidisciplinary management as soon as the diagnosis is confirmed.

## Conclusions

This case highlights that HD can initially present with predominant cognitive decline and subtle, asymmetric motor findings, emphasizing that early non-motor symptoms should not be overlooked. Careful neuropsychological assessment, combined with detailed family history, is essential to raise suspicion and guide timely genetic testing. Genetic confirmation not only establishes a definitive diagnosis but also enables targeted genetic counseling, accurate family risk assessment, and planning for supportive interventions. While no disease-modifying therapy currently exists, early recognition allows prompt initiation of multidisciplinary management, including cognitive, psychiatric, and rehabilitative support, which can help optimize quality of life, anticipate complications, and improve patient outcomes. Such presentations contribute to greater clinical awareness and may facilitate earlier identification of HD in similar cases.
